# Biomarker Potential of Interleukin-6 in Differentiating Necrotizing Enterocolitis from Late-Onset Sepsis in Neonates Born Preterm

**DOI:** 10.1016/j.jpedcp.2024.200138

**Published:** 2025-01-02

**Authors:** Ceren Imren, Vivian de Groot, Claudia M.G. Keyzer-Dekker, Irwin K.M. Reiss, Sten P. Willemsen, Marijn J. Vermeulen, H. Rob Taal

**Affiliations:** 1 Department of Pediatric Surgery, Erasmus MC Sophia Children's Hospital, Rotterdam, the Netherlands; 2 Division of Neonatology, Department of Neonatal and Pediatric Intensive Care, Erasmus MC Sophia Children's Hospital, Rotterdam, the Netherlands; 3 Department of Biostatistics, Erasmus MC University Medical Center Rotterdam, Rotterdam, the Netherlands

**Keywords:** cytokines, inflammation, diagnostics, sepsis, gastrointestinal

## Abstract

**Objective:**

Interleukin-6 (IL-6) is an early biomarker for sepsis and necrotizing enterocolitis (NEC). We assessed IL-6's ability to differentiate between late-onset sepsis (LOS) and NEC and between medical and surgical NEC.

**Study design:**

This retrospective cohort study included infants born preterm (birth weight <1500 g, gestational age <32 weeks) with ≥1 episodes of suspected late-onset sepsis (sLOS) between 2018 and 2023. Plasma IL-6 levels at sLOS onset were analyzed. Infants were grouped into (1) control (no sepsis/NEC), (2) LOS (culture negative/positive sepsis), or (3) NEC (medical/surgical), on the basis of the greatest classification of their observed episodes. IL-6's predictive value (alone and in combination with C-reactive protein) for sLOS outcomes was assessed with receiver operating characteristic analysis, with the area under the curve (AUC) quantifying its discriminative quality.

**Results:**

sLOS was observed in 421 infants (670 episodes); 131 (31%) had no LOS or NEC, 225 (53%) had LOS without NEC, and 65 (15%) had NEC. Median IL-6 values significantly differed between all groups, with highest in infants with NEC. The odds of NEC over LOS increased by a factor of 1.53 (95% CI 1.42-1.65, *P* < .001) for every doubling of IL-measurements. IL-6 was not associated with the odds of surgical NEC compared with medical NEC. IL-6's ability to distinguish NEC from LOS was moderate (AUC 0.73). IL-6 combined with C-reactive protein (AUC 0.64) showed poor discriminative ability.

**Conclusions:**

Although elevated IL-6 levels are associated with greater odds of having NEC instead of LOS, the moderate predictive value suggests that IL-6 alone may not be sufficient for accurate early diagnosis or differentiation.

Necrotizing enterocolitis (NEC) is a leading cause for critical illness in infants born preterm with neonatal treatment, presenting as an acute, life-threatening inflammation and ischemia of the immature intestinal tract.^1^ Diagnosing NEC remains challenging, as early clinical signs are often nonspecific and can resemble those of sepsis. NEC affects approximately 7%-10% of infants born preterm with a birth weight ≤1000 g, whereas sepsis occurs at least twice as often.^2^ Sepsis and NEC exhibit complex interactions, with late-onset neonatal sepsis (LOS) increasing the risk of NEC development, whereas NEC can serve as the underlying cause of neonatal sepsis.^3^, ^4^, ^5^ Approximately 40%-60% of NEC cases present concurrently with sepsis, challenging their distinction as the result of similar clinical symptoms.^6^ Timely differentiation between these 2 conditions is essential, as their management significantly differs in antibiotic therapy and feeding strategy. Early and efficient treatment is crucial for outcome and prognosis in both diseases. An easily accessible and well-differentiating biomarker could avoid unnecessary delay of diagnosis.

In the ongoing search for such a tool, some biomarkers have been identified to indicate either sepsis or NEC. Interleukin-6 (IL-6), a proinflammatory cytokine, is a proven biomarker for the early detection of neonatal sepsis. It exhibits a more rapid response compared with other biomarkers like C-reactive protein (CRP) and procalcitonin.^7^ Previously, studies with a small sample size (<25 infants with NEC) have demonstrated elevated IL-6 levels in infants with NEC compared with healthy controls^8^ and greater levels in surgical NEC compared with medical NEC.^9^ The aim of this study is to investigate the utility of IL-6 as a biomarker for differentiating between sepsis and NEC, and between medical and surgical NEC cases, in a large cohort of infants with suspected LOS. We hypothesized that IL-6 values would significantly differ between sepsis and NEC, and between medical and surgical NEC, adding new valuable insights for timely and tailored clinical interventions to the existing knowledge.

## Study Design

### Study Design and Population

This retrospective cohort study was conducted at the Erasmus MC University Medical Center-Sophia Children's Hospital Rotterdam, a level IV neonatal intensive care unit (NICU). Inclusion criteria for the study population were (1) gestational age <32 weeks and/or birth weight <1500 g and (2) admission to the NICU between January 1, 2018, and December 31, 2023. Infants were excluded if they had been referred to our hospital later than 48 hours after birth, to ensure consistent early clinical data collection. For the current study, the eligible study population was screened for all neonates with episodes of suspected late-onset neonatal sepsis (sLOS) during their admission to the NICU, for whom blood cultures and IL-6 values were assessed as part of a sepsis work-up.

### Ethical Approval

This study was part of the ongoing Risk NEC study (MEC-2013-409), which was reviewed by the local ethical board of the Erasmus MC, University Medical Center. It was designed to identify risk factors and assess outcomes related to NEC. On the basis of the retrospective and observational nature of the study, the local ethical board waived the need for explicit informed consent for this specific study. However, it is standard practice in our unit to request general parental consent for the use of data in future medical research for all admitted infants. Infants whose parents do not consent are excluded from future studies.

### Definitions

LOS (>72 hours after birth) diagnosis was established using the criteria of the *Eunice Kennedy Shriver* National Institute of Child Health and Human Development Neonatal Research Network.^10^ LOS was defined as either blood culture positive (gram positive or gram negative, with intention to treat with antibiotics for >5 days), or blood culture negative (with a CRP level >10 mg/L within 2 days after the blood culture was taken, intention to treat with antibiotics for >5 days, and clinical symptoms of sepsis [Appendix; available at www.jpeds.com, Table I], both without symptoms suggestive for NEC, assessed by an attending physician). NEC was diagnosed on the basis of clinical and radiologic findings (Appendix; available at www.jpeds.com, Table II), as well as on surgical and pathologic reports when available. On the basis of treatment, infants were categorized as having either medical or surgical NEC. Surgical NEC was defined as NEC with an indication for surgery within 7 days after diagnosis. sLOS episodes were classified as (1) no sepsis or NEC (negative blood culture, antibiotics ceased <96 hours), (2) LOS, or (3) NEC, which was considered the highest classification. NEC and sepsis could occur simultaneously, in which case an episode was classified as NEC. A single infant could demonstrate multiple episodes. IL-6 levels associated with episodes after the occurrence of NEC were not included. Infants were ordinally categorized into (1) control group (no sepsis or NEC), (2) sepsis group (LOS), or (3) NEC group (medical and surgical NEC), on the basis of the greatest classification of any episode they demonstrated.

**Table I:** Patient characteristics

Characteristics	Control∗ n = 131	LOS∗ n = 225	NEC∗ n = 65	*P* value†
Sex, male	72 (55)	133 (59)	37 (57)	.74
Multiple births	24 (18)	45 (20)	14 (22)	.86
Gestational age, wk + d	27 + 5 [26 + 2 to 29 + 0]	26 + 5 [25 + 1 to 28 + 6]	25 + 6 [25 + 0 to 27 + 0]	.02‡
Birth weight, g	980 [785-1215]	840 [680-1045]	825 [735-1012]	.04‡
Birth weight, Fenton z sore	0.2 [–0.8 to 0.7]	−0.2 [–1.1 to 0.5]	0.3 [–0.1 to 0.7]	.002§
Mode of delivery, cesarean	91 (69)	148 (66)	39 (60)	.42
			n = 1 missing	.07
Antenatal steroids	83 (63)	151 (67)	35 (55)	
	n = 1 missing	n = 4 missing	n = 1 missing	.45
Apgar score, 5 min	8 [7-9]	8 [7-9]	7 [6-8]	
Respiratory support¶				
Mechanical ventilation	79 (60)	169 (75)	62 (95)	<.001∗∗
Ventilation days	3 [0-14]	7 [1-19]	13 [5-21]	<.001∗∗
Type of feeding				.99
Only human milk	90 (69)	154 (68)	43 (66)	
Only formula	2 (2)	3 (1)	1 (2)	
Combination	39 (30)	68 (30)	21 (32)	
Number of sLOS episodes	1 [1-2]	1 [1-2]	2 [1-3]	.002‡
NEC				
Medical NEC/surgical NEC			18 (28)/47 (72)	
NEC totalis			11 (17)	
Duration of NICU admission,†† d	41 [19-66]	49 [20-78]	47 [11-97]	.31
Mortality during NICU admission	12 (9)	34 (15)	27 (42)	<.001‡‡

Data are presented as No. (%) or median [IQR].

∗ Group assignment on the basis of the greatest observed episode.

† χ^2^ tests for categorical data, and Kruskal-Wallis tests for continuous data; post-hoc Bonferroni tests for multiple testing. A *P* value of <.05 was considered statistically significant.

‡ LOS-control and NEC-control.

§ LOS-control and LOS-NEC.

¶ During NICU admission.

∗∗ Control-LOS, control-NEC, and LOS-NEC.

†† Duration from admission until discharge or death (whichever was first).

‡‡ Control-NEC, LOS-NEC.

**Table II:** Blood culture outcomes

Sepsis type	LOS n = 338	NEC n = 65	*P* value∗
No sepsis	–	9 (14)	
IL-6 values, pg/mL	–	135 (36-415)	
Culture-negative sepsis	146 (43)	19 (29)	
IL-6 values, pg/mL	199 (90-383)	1467 (206-8062)	<.001
Gram-positive sepsis	148 (44)	20 (31)	
IL-6 values, pg/mL	285 (82-1034)	330 (109-1951)	.321
Coagulase-negative staphylococci	97 (66)	14 (45)	
*Staphylococcus aureus*	30 (20)	2 (6)	
*Enterococcus faecalis*	12 (8)	3 (10)	
Group B streptococci	5 (3)	–	
*Bacillus cereus*	2 (1)	–	
*Streptococcus mitis*	1 (1)	–	
*Candida*	1 (1)	1 (3)	
Gram-negative sepsis	44 (13)	17 (26)	
IL-6 values, *pg*/*mL*	2574 (327-37 548)	2204 (878-17 936)	.923
*Escherichia coli*	21 (48)	10 (59)	
*Enterobacter cloacae*	9 (20)	3 (18)	
*Pseudomonas aeruginosa*	5 (11)	1 (6)	
*Klebsiella pneumoniae*	4 (9)	3 (18)	
*Serratia marcescens*	3 (7)	–	
*Citrobacter freundii*	2 (5)	–	

Data are presented as No. (%) or as median (IQR).

∗ Mann-Whitney *U* tests were performed. A *P* of < .05 was considered statistically significant.

### Patient Characteristics

Patient characteristics were retrieved from the Risk NEC study database, including sex, gestational age, birth weight, birth weight for age z score (Fenton and Kim^11^), mode of delivery, multiple births, antenatal corticosteroid therapy (full course, eg, at least 48 hours of therapy), type of feeding before sLOS episode, respiratory support (mechanical ventilation), NEC, NEC treatment (medical or surgical), and NEC totalis. NEC totalis was defined as necrosis of the majority of small intestines without reasonable curative treatment options seen at a laparotomy, leading to an open-close procedure.^12^

### Blood Cultures and Biomarkers

In January 2018, our NICU implemented a local guideline for using heart rate variability (HRV) monitoring in combination with the determination of inflammatory biomarkers as an early warning system for LOS.^13^ When the HRV monitoring system warns for possible sepsis signs, inflammatory biomarkers (including IL-6, CRP, and procalcitonin) are obtained as part of standard care. If these biomarkers are increased, a blood culture is drawn and antibiotics are started. At all times, strong clinical suspicion of sepsis immediately led to drawing a blood culture and administration of antibiotics. Onset of a sLOS episode was defined as the moment of drawing a blood culture. For the current study, only IL-6 values were analyzed and sLOS episodes were excluded if no IL-6 value was available. Blood culture results from the moment of sepsis suspicion were retrieved from the Department of Microbiology. Serum IL-6 values (in pg/mL) were measured by the department of Clinical Chemistry, using Electro-Chemi Luminescent Immuno Assay tests (E801, Cobas 8000 system; Roche Diagnostics). The limits of detection were 1.5 to 10 000 pg/mL, extendable with further dilution. Values were extracted from the laboratory data system. Each sLOS episode was matched with the corresponding IL-6 value measured at the onset of the episode (one IL-6 value per episode).

### Statistical Analyses

To conduct pairwise comparisons of categorical data between outcome groups (control, LOS, NEC), χ^2^ tests were performed. For continuous data, Kruskal-Wallis or Mann-Whitney *U* tests were performed, depending on the number of groups involved in the comparison. To analyze the predictive value of IL-6 for sLOS episode outcomes, a receiver operating characteristic analysis was conducted. The area under the curve (AUC) was assessed to quantify the overall discriminative ability of IL-6 alone. An AUC score of 0.50-0.69 was considered poor, 0.70-0.79 was considered moderate, and ≥0.80 was considered excellent in terms of discriminative value. The Youden index was used to calculate optimal cut-off points. To assess the combined discriminative value of IL-6 and CRP, predicted probabilities from logistic regression were used in additional receiver operating characteristic analyses, on the basis of measurements taken at the same time point. Calculating cut-off points was not feasible in these analyses because of the use of predicted probabilities. In patients who experienced multiple episodes of sLOS (a single IL-6 value per episode), multiple IL-6 data were available (repeated episodes). Therefore, to evaluate the discriminative potential of IL-6 levels in classifying sLOS episodes, a continuation ratio mixed model (CRMM) appropriate for repeated and ordinal data was constructed.^14^ A CRMM estimates the conditional odds of progressing to a greater category vs remaining in the current category. In this context, the OR represents the odds of transitioning (1) from the control group to either LOS or NEC and (2) from LOS to NEC, given that a patient has at least reached the LOS category, for every doubling of IL-6 levels. The model’s goodness-of-fit was assessed with a log-likelihood ratio χ^2^ test. To analyze the value of IL-6 in differentiating between medical and surgical NEC, a logistic regression was performed. Both models were adjusted for sex, gestational age, and birth weight z score. Outcomes are presented as ORs with a 95% CI and a *P* value. Analyses were performed on raw or binary log-transformed data, depending on normality. A *P* value less than .05 was considered statistically significant. The Bonferroni correction was applied for multiple testing (maximum of 2 independent tests). Statistical analyses were performed using SPSS, version 28.0 (IBM SPSS Statistics for Windows). R Statistics (version 4.3.1; R Core Team 2023) was used for the CRMM analysis, using the *GLMMadaptive* (Rizopoulos, 2023), *dplyr* (Wickham, 2023), *ordinal* (Christensen, 2023), and *ggplot2* packages (Wickham, 2023).

## Results

### Patient Characteristics

**Figure 1:**
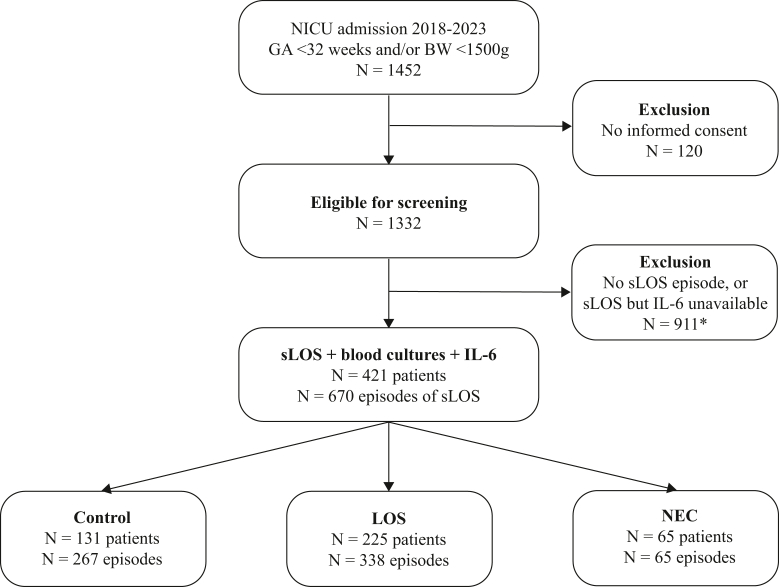
Flowchart of inclusion and episode classification. ∗Including 20 patients with NEC. *BW*, birth weight; *GA*, gestational age.

The eligible study population comprised 1332 infants, of whom 421 (32%) showed ≥1 episodes of sLOS with blood cultures and IL-6 measurements available (Figure 1). In total, we included 640 episodes. Median number of sLOS episodes per infant was 1 [range 1-7]; 258 infants had a single episode and 163 had ≥2 episodes. Ordinal categorization resulted in 131 infants (31%) with no proven LOS or NEC (control group), 225 (53%) with LOS, and 65 (15%) with NEC. Patient characteristics for each group are summarized in Table I. Median gestational age differed between all groups, with infants who had NEC being born at lowest gestational age. Infants in the LOS group had a lower median birth weight for gestational age compared with infants in the control or NEC group. The need for invasive respiratory support and of ventilation days differed between all groups. Mechanical ventilation was most common (95%) and longest (median of 13 days, range 0-83) among infants with NEC. Mortality was greatest in the NEC group.

### IL-6 per Episode Type

**Figure 2:**
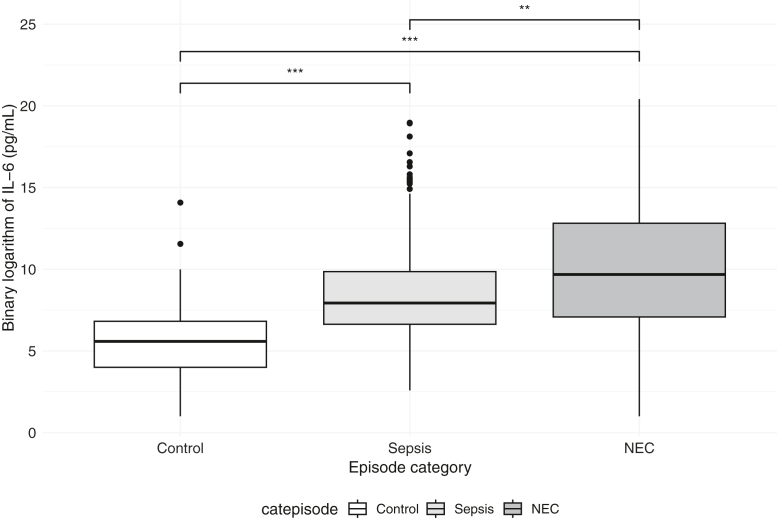
IL-6 levels in picogram per milliliter (pg/mL) at moment of sLOS, per episode category. Data are presented as binary log-transformed data because of the wide range in IL-6 levels. Median IL-6 for controls: 48 pg/mL (IQR 16-113 pg/mL), for sepsis: 244 pg/mL (IQR 99-935 pg/mL), and for NEC: 819 pg/mL (131-7542 pg/mL). The outliers seen in the LOS group were mostly attributable to gram-negative sepsis cases (in 14/19 cases). ∗∗*P* = .01 ∗∗∗*P* < .001.

sLOS episodes were categorized into 267 control episodes (267/640, 41%), 338 LOS (53%), and 65 NEC (10%). Figure 2 shows the plasma IL-6 levels at the moment of sLOS per episode type. The median IL-6 level for controls was 48 pg/mL (IQR 16-113 pg/mL), 244 pg/mL (IQR 99-935 pg/mL) for LOS, and 819 pg/mL (IQR 131-7,542 pg/mL) for NEC episodes. Median IL-6 values significantly differed between all episode types, with the highest values in NEC cases. The median IL-6 value in medical NEC was 565 pg/mL (IQR 67-5,747 pg/mL) and in surgical NEC 1021 pg/mL (IQR 143-7929 pg/mL); these did not significantly differ (*P* = .453). No statistically significant differences in IL-6 values were observed among infants with surgical NEC with and without NEC totalis (1572 vs 679 pg/mL, *P* = .06).

### IL-6 per Blood Culture Outcome

Blood culture assessment showed negative blood cultures in 146 of 338 (43%) LOS episodes, gram-positive blood cultures in 148 (44%), and gram-negative cultures in 44 (13%). Among NEC episodes, 19 of 65 (29%) blood cultures were classified as culture-negative sepsis, 20 (31%) were gram positive, and 17 (26%) were gram negative. Nine infants had NEC without sepsis (14%). Median IL-6 values significantly differed between culture-negative LOS and culture-negative NEC (199 vs 1467 pg/mL; *P* < .001, Table II). Isolated organisms are listed in Table II. No statistically significant differences in IL-6 values between LOS and NEC were observed in gram-positive (285 vs 330 pg/mL, *P* = .321) and gram-negative sepsis (2547 vs 2204 pg/mL, *P* = .923). Among infants with LOS, IL-6 values were significantly greater in gram-negative sepsis compared with gram-positive sepsis (2574 vs 285 pg/mL, *P* ≤ .001). In infants with NEC, this difference in IL-6 values between gram-negative and gram-positive blood sepsis was not statistically significant (2204 vs 330 pg/mL, *P* = .297).

### IL-6 for Differentiating NEC From LOS and Controls

**Figure 3:**
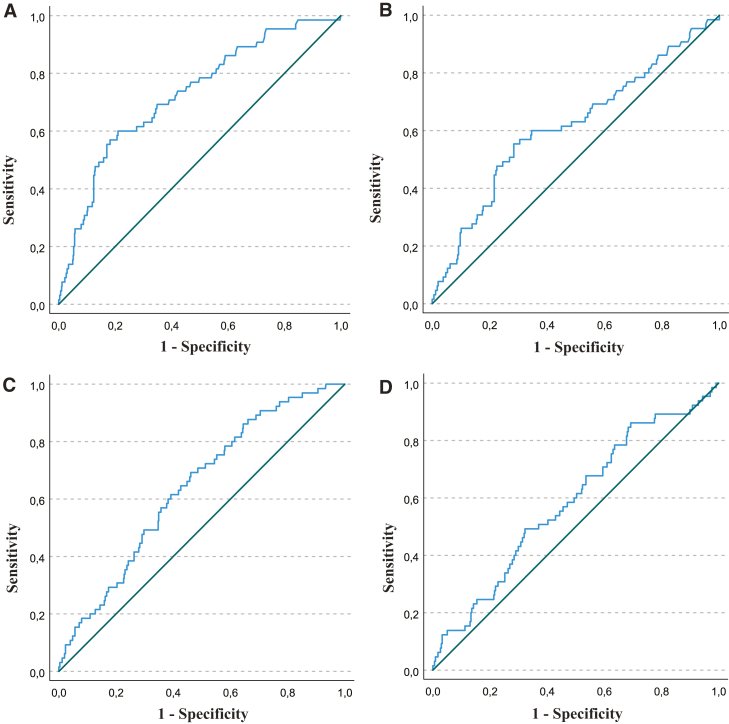
**A,** Receiver operating characteristic curve for IL-6 values to differentiate NEC from other sLOS episodes (control + LOS). AUC: 0.73 (95% CI 0.66-0.79). Calculated cut-off point: 441 pg/mL, sensitivity: 60%, specificity: 79%. **B,** ROC curve for IL-6 values to differentiate NEC from LOS. AUC: 0.61 (95% CI 0.54-0.69). Calculated cut-off point: 675 pg/mL, sensitivity: 55%, specificity: 72%. **C,** ROC curve for IL-6 and CRP combined to differentiate NEC from other sLOS episodes (control + LOS). AUC: 0.64 (95% CI 0.58-0.71). Sensitivity: 69%, specificity: 54%. **D,** ROC curve for IL-6 and CRP combined to differentiate NEC from LOS. AUC: 0.58 (95% CI 0.51-0.66). Sensitivity: 49%, specificity: 68%. *ROC*, receiver operating characteristic.

The AUC for IL-6 alone to differentiate between NEC and all other sLOS episodes (control and LOS combined, n = 65 vs n = 605) was 0.73 (95% CI 0.66-0.79, *P* < .001) (Figure 3, A), indicating a moderate discriminative ability. The optimal cut-off point for IL-6 was calculated at 441 pg/mL, with a sensitivity of 60% and a specificity of 79%. The area under the curve (AUC) for IL-6 alone to differentiate between LOS and NEC was 0.61 (95% CI 0.54-0.69, *P* = .005) (Figure 3, B), indicating poor discriminative ability. The optimal cut-off point for IL-6 was established at 675 pg/mL, at which point IL-6 reached a sensitivity of 55% and a specificity of 72%.

The combined discriminative ability of IL-6 and CRP for differentiating NEC from all other sLOS episodes showed an AUC of 0.64 (95% CI 0.58-0.71) (Figure 3, C), indicating a poor discriminative ability. Sensitivity was calculated at 69% and specificity at 54%. For differentiating NEC from LOS, the AUC was 0.58 (95% CI 0.51-0.66) (Figure 3, D), indicating poor discriminative capabilities. Sensitivity was calculated at 49% and specificity at 68%.

### Predictive Value of IL-6 for NEC

Outcomes of the CRMM are summarized in Table III. Binary logarithmically transformed IL-6 was associated with increased odds of having NEC vs LOS (OR 1.53, 95% CI 1.42-1.65). This means the odds of having NEC over LOS increased by a factor of 1.53 for every doubling of IL-6 measurements. Gestational age showed a weak negative association: a 1-unit increase in gestational age (ie, 1 week) reduces the odds of having NEC instead of LOS (OR 0.90, 95% CI 0.82-0.99, *P* = .03). Sex and birth weight did not influence the conditional odds of being in the LOS group vs in the NEC group, as they were not significant. There was no significant association in binary logarithmically transformed IL-6 levels for the odds of having surgical NEC compared with medical NEC (OR 1.08, 95% CI 0.87-1.32, *P* = .44).

**Table III:** Results of the continuation ratio model: (episode outcome > LOS vs outcome = LOS)

Model variable	b	SE(b)	OR	95% CI OR	*P* value
Intercept	−3.57	1.25			
Cohort ≥ sepsis	3.67	0.27			
Ln_2_(IL-6)	0.43	0.04	1.53	1.42-1.65	<.001
Sex	−0.05	0.19	0.95	0.65-1.39	.80
Male = 1					
Birth weight, z score	−0.03	0.10	0.97	0.80-1.18	.78
Gestational age, wk + d	−0.10	0.05	0.90	0.82-0.99	.03

*b*, estimated logit coefficient; *ln*_*2*_, binary logarithmically transformed.

All episodes (n = 670) were included in the analysis.

Log-likelihood ratio χ^2^ test for goodness of fit of the full model compared with nested model (only Ln_2_[IL-6] included): 4.42, *P* = .22.

## Discussion

Our results show significantly greater IL-6 values in cases of NEC compared with LOS, especially when blood cultures are negative (clinical sepsis without symptoms suggestive for NEC). Elevated IL-6 levels are associated with greater odds of NEC compared with LOS and the discriminative value to distinguish NEC from LOS or control episodes is moderate. Combining IL-6 with CRP values did not enhance IL-6's discriminative power. There were no significant differences in IL-6 levels between medical and surgical NEC cases, nor between surgical NEC cases with and without NEC totalis.

Elevated IL-6 levels in patients with NEC have been described previously in studies with small sample sizes (n < 25), comparing them either with healthy infants born preterm or with infants experiencing feeding intolerance, culture-positive, or suspected LOS.^8^^,^^9^^,^^15^, ^16^, ^17^ Only Wisgrill et al. specifically investigated the ability of IL-6 to distinguish between proven culture-positive LOS and NEC in infants born preterm, reporting moderate discriminatory performance.^9^ Our study population most closely resembles that of Harris et al, as both studies included infants born preterm with suspected sepsis and measured IL-6 at the onset thereof.^16^ However, the outcomes of their study are less comparable with ours because of their small study sample (n = 62) and being conducted in the previous century, which may limit generalizability and reliability of measurements. HRV monitoring was not yet implemented at the time; their onset of suspected sepsis might be later than ours. In addition, their IL-6 measurements were limited to a maximum of 160 pg/mL, whereas modern technology allows for measurements without such limitations.

Wisgrill et al demonstrated significantly greater IL-6 values—measured at the time of NEC diagnosis—in surgical NEC compared with medical NEC cases, with excellent discriminatory ability (AUC 0.931).^9^ However, they included only 12 surgical and 12 medical NEC cases, which may influence the robustness of their findings. In our clinical study, IL-6 was measured at the onset of suspected illness, which is usually earlier than the moment of definite NEC diagnosis. This timing difference may explain the lower discriminating value found in the present study, suggesting that although it is the appropriate timing to adjust the clinical policy, measuring IL-6 too early in the inflammatory process might not accurately capture the cytokine response. This may also explain the lack of significant association of IL-6 with NEC totalis. Since the diagnosis NEC totalis can only be made perioperatively, we did not aim to predict the condition but sought associations between IL-6 and disease severity. Only one study previously investigated this association. Bhatia et al assessed preoperative levels of cytokines including IL-6, interleukin-8 (IL-8), and tumor necrosis factor-α in surgical NEC with and without NEC totalis, reporting significantly greater IL-8 values in NEC totalis cases.^18^ However, the small sample size (n = 4 with and n = 8 without NEC totalis) limits the interpretability of their findings.

Franz et al found that the predictive value of IL-8 for bacterial infections in newborns was enhanced when combined with CRP.^19^ In contrast, our study showed that CRP did not enhance the discriminative ability of IL-6 for diagnosing NEC. This might suggest that although CRP is a common inflammatory marker, it does not provide additional discriminatory value for diagnosing NEC in this specific population of neonates born preterm with a sLOS.

IL-6 levels were greatest in cases of gram-negative sepsis in patients with LOS, a finding previously described in a smaller cohort by Kurul et al.^20^ A similar trend was observed in infants with NEC, although it did not reach statistical significance. The outcomes of this analysis suggest that IL-6 might serve as a marker for gram-negative organisms more effectively than for gram-positive ones. This is consistent with well-established immunological principles regarding the immune response to gram-negative infections. Lipopolysaccharides in the outer membrane of gram-negative bacteria are known to strongly induce cytokine production, including IL-6, through activation of toll-like receptors (primarily TLR4) on immune cells.^21^ Gram-positive bacteria lack lipopolysaccharides, which may explain the difference in IL-6 levels.

In contrast to other recent studies, we also included culture-negative LOS to accurately reflect the NICU population. Culture-negative LOS is commonly diagnosed in clinical practice^22^ but can be difficult to interpret and may introduce bias. It is important to note that blood sampling in infants born preterm has practical limitations, such as the lack of routine second blood cultures, which is standard in adult diagnostics. It remains unclear whether these cases are attributable to false-negative blood cultures or represent a different condition entirely. Post-hoc analysis of our data revealed a mortality of 19% in the culture-negative LOS group, compared with 12% for gram-positive LOS and 38% for gram-negative LOS, indicating that infants with culture-negative LOS were not necessarily less ill.

### Strengths and Limitations

Our study's main strength is its relatively large sample size, representing the largest cohort of 421 patients, of whom 65 had NEC, to date. This provided the statistical power necessary for advanced models to assess the association between IL-6 levels and clinical outcomes. The inclusion of a substantial control group enhanced the reliability of our comparisons. Another strength is the study design, with timing of IL-6 measurements at the onset of suspected illness. HRV monitoring in our hospital alerts clinicians on the basis of changes in HRV, enabling sepsis work-up at an early stage, sometimes before specific clinical signs manifest. A previous study showed that this approach can identify sepsis sooner and reduce its severity, without leading to increased numbers of blood cultures or unnecessary antibiotic use.^13^ Our study's rate of proven LOS and NEC rate is consistent with those reported in other studies on infants born preterm or with low birth weight.^23^, ^24^, ^25^ This timing of measurements offers an accurate reflection of IL-6's potential as an early biomarker for distinguishing between conditions. However, this also presents limitations, as the short half-life and rapid and short peak of IL-6 suggests a limited window of opportunity to best measure its values. In addition, not all NICU centers use HRV monitoring, which may limit the generalizability of our findings. Other limitations that warrant consideration include the retrospective design, which increases the risk of missing data. For instance, among the 911 infants who were excluded because of missing IL-6 values or blood cultures were 20 infants with NEC, reducing the number of NEC cases included in the analyses and potentially introducing selection bias. The Youden Index, which was used to calculate optimal cut-off point for IL-6, assigns equal weight to false-positive and false-negative cases, which may not be entirely appropriate in this setting. Therefore, the calculated cut-off points should be interpreted with caution.

### Future Research

Although elevated IL-6 levels were associated with greater odds of NEC, the moderate predictive value observed suggests that IL-6 alone may not be sufficient for accurate early diagnosis. However, IL-6 could still be a valuable component of a multimarker approach, combined with other clinical and biochemical indicators other than CRP. Benkoe et al identified IL-6, as well as IL-8 and IL-10, as promising cytokines in the diagnosis of NEC.^8^ Further research could include prospective, longitudinal monitoring of these cytokines combined. For instance, measuring markers at multiple time points may lead to the development of a predictive model to distinguish between medical and surgical NEC, as well as NEC totalis. This could have therapeutic implications, such as guiding anti-inflammatory or surgical strategies.

## Conclusions

Although elevated IL-6 levels are associated with greater odds of NEC, the moderate predictive value suggests that IL-6 alone may not be sufficient for accurate early diagnosis. This emphasizes the need for further research into multimarker approaches, combining IL-6 with other clinical or biochemical markers. The findings from our study contribute to the growing body of evidence on the role of IL-6 in diagnosing neonatal sepsis and NEC, underscoring the importance of early and accurate diagnosis in improving outcomes for this vulnerable population.

## CRediT authorship contribution statement

**Ceren Imren:** Writing – original draft, Visualization, Validation, Software, Resources, Project administration, Methodology, Investigation, Formal analysis, Data curation, Conceptualization. **Vivian de Groot:** Writing – review & editing, Resources, Investigation, Data curation, Conceptualization. **Claudia M.G. Keyzer-Dekker:** Writing – review & editing. **Irwin K.M. Reiss:** Writing – review & editing. **Sten P. Willemsen:** Software, Formal analysis. **Marijn J. Vermeulen:** Writing – review & editing, Supervision, Methodology, Conceptualization. **H. Rob Taal:** Writing – review & editing, Validation, Supervision, Resources, Methodology, Conceptualization.

## Declaration of Competing Interest

The authors have no conflicts of interest to disclose.
