# Supplementary material for: Biomarker Potential of Interleukin-6 in Differentiating Necrotizing Enterocolitis from Late-Onset Sepsis in Neonates Born Preterm
Source: J Pediatr Clin Pract. 2025 Jan 2;15:200138. doi: 10.1016/j.jpedcp.2024.200138 (PMC11824627; doi:10.1016/j.jpedcp.2024.200138)
Supplement: Appendix A [file mmc1.docx]

**Appendix A: Definitions**

| ***Clinical symptoms*** |
| --- |
| Irritability |
| Lethargy |
| Feeding intolerance |
| Respiratory distress |
| Temperature instability |
| Bradycardia/tachycardia |
| Prolonged capillary refill (>2 seconds) |
| Apnea |

Table 1. Clinical symptoms of late onset sepsis (LOS) [1].

| ***Clinical symptoms*** | ***Radiological symptoms (abdominal x-ray)*** |
| --- | --- |
| Distended, tender abdomen | Ileus |
| Gastric retention | Intestinal dilatation |
| Bloody stool | Pneumatosis intestinalis |
| Signs of peritonitis | Portal venous gas |
| Lethargy | Pneumoperitoneum |
| Temperature instability | Ascites |
| Bradycardia/tachycardia |  |
| Apnea |  |
| Respiratory and/or metabolic acidosis |  |

Table 2. Clinical and radiological symptoms of necrotizing enterocolitis (NEC) [2].

1. Boghossian, N.S., et al., *Late-onset sepsis in very low birth weight infants from singleton and multiple-gestation births.* J Pediatr, 2013. **162**(6): p. 1120-4, 1124 e1.

2. Walsh, M.C. and R.M. Kliegman, *Necrotizing enterocolitis: treatment based on staging criteria.* Pediatr Clin North Am, 1986. **33**(1): p. 179-201.
